# Evaluation and Modeling of the Fatigue Damage Behavior of Polymer Composites at Reversed Cyclic Loading

**DOI:** 10.3390/ma12111727

**Published:** 2019-05-28

**Authors:** Ilja Koch, Gordon Just, Martin Brod, Jiuheng Chen, Audrius Doblies, Aamir Dean, Maik Gude, Raimund Rolfes, Christian Hopmann, Bodo Fiedler

**Affiliations:** 1 Institute of Lightweight Engineering and Polymer Technology (ILK), Technische Universität Dresden, Holbeinstr. 3, 01307 Dresden, Germany; gordon.just@tu-dresden.de (G.J.); maik.gude@tu-dresden.de (M.G.); 2 Institute of Structural Analysis (ISD), Leibniz Universität Hannover, Appelstr. 9A, 30167 Hannover, Germany; m.brod@isd.uni-hannover.de (M.B.); a.dean@isd.uni-hannover.de (A.D.); r.rolfes@isd.uni-hannover.de (R.R.); 3 Institute for Plastics Processing (IKV), RWTH Aachen University, Seffenter Weg 201, 52074 Aachen, Germany; jiuheng.chen@ikv.rwth-aachen.de (J.C.); hopmann@ikv.rwth-aachen.de (C.H.); 4 Institute for Polymers and Composites (IPC), Technische Universität Hamburg-Harburg, Denickestr. 15, 21073 Hamburg, Germany; audrius.doblies@tuhh.de (A.D.); fiedler@tuhh.de (B.F.)

**Keywords:** FRP, fatigue, block-loading, load reversal, residual stresses, modeling

## Abstract

Understanding the composite damage formation process and its impact on mechanical properties is a key step towards further improvement of material and higher use. For its accelerated application, furthermore, practice-related modeling strategies are to be established. In this collaborative study, the damage behavior of carbon fiber-reinforced composites under cyclic loading with load reversals is analyzed experimentally and numerically. The differences of crack density evolution during constant amplitude and tension-compression block-loading is characterized with the help of fatigue tests on cross-ply laminates. For clarifying the evolving stress-strain behavior of the matrix during static and fatigue long-term loading, creep, and fatigue experiments with subsequent fracture tests on neat resin samples are applied. The local stress redistribution in the composite material is later evaluated numerically using composite representative volume element (RVE) and matrix models under consideration of viscoelasticity. The experimental and numerical work reveals the strong influence of residual stresses and the range of cyclic tension stresses to the damage behavior. On the microscopic level, stress redistribution dependent on the mean stress takes place and a tendency of the matrix towards embrittlement was found. Therefore, it is mandatory to consider stress amplitude and means stress as inseparable load characteristic for fatigue assessment, which additionally is influenced by production-related and time-dependent residual stresses. The phenomenological findings are incorporated to a numerical simulation framework on the layer level to provide an improved engineering tool for designing composite structures.

## 1. Introduction

During mechanical loading of fiber-reinforced plastics (FRP), damage phenomena occur with increasing stress levels and duration, which can lead to material degradation. The damage is determined by the inhomogeneity of the material across different scales, which is characterized microscopically by the characteristic lengths of filament and matrix, mesoscopically by the thickness of the diverse oriented layers, and macroscopically by the spatial laminate expansion. Analogous to this geometrically motivated scale definition, the damage phenomena can be classified into micro, meso, and macro damage. On the macroscopic scale, composite material damage can be observed as stiffness and strength degradation through the formation of damage on the meso, or layer, level. The damage phenomena, such as delamination, fiber-breaking and matrix cracks, are formed statistically distributed in the laminate and are initiated by microscopic phenomena in the matrix and fiber-matrix interphase. The characteristic of damage caused by cyclic loading is determined mainly by mean stress and stress amplitude and thus by sign and orientation of the varying load vector with respect to the fiber direction. Additional factors influencing the crack initiation and growth are the load frequency and load sequence, environmental conditions (temperature, humidity, etc.), manufacturing process, and material configuration.

### 1.1. State of the Art

Recently an increasing attention is paid on understanding load reversals and their detrimental effect on damage accumulation [1,2,3,4,5,6].

Gamstedt et al. [3] studied the effects caused by load reversal on a ${\left[0/90\right]}_{s}$ laminate. Therein, in a cyclic loading condition with load reversal, in regions of tension-induced delaminations, it is observed that a subsequent compression loading leads to local instability failure and fiber kinking. Furthermore, cyclic loading with load reversal—compared to pure tension or compression loading—leads to damage with a higher density of matrix cracks, accumulated interface failure as well as to a higher amount of micro-damage in the adjacent plies.

Quaresimin et al. [4] analyzed the influence of load ratio and load biaxiality on the damaging process of glass fiber-reinforced epoxy (GFRP) tubes experimentally for three different load ratios and biaxiality ratios, respectively. They found that in the case of pure transverse loading of the embedded 90${}^{\circ }$-layer, fully reversed tests at R=−1 lead to earlier crack initiation and the highest crack growth rates compared to the other tested load ratios.

These findings are supported by results for carbon fiber-reinforced (CFRP) cross-ply laminates tested at four different load ratios published by the authors of this publication in earlier work [6,7]. The fatigue experiments on CFRP strip specimens revealed a more pronounced crack growth for load ratios R<0, compared to experiments with R>0.

However, the possible reasons of the accelerated damage growth under reversed loading are still an open research topic and the driving mechanisms on the microscale have to be analyzed to derive physical-based macroscopic formulations of the damage growth.

Over the last few years, a great effort has been devoted not only to experimental investigations of fatigue damage of composites but also to the development of numerical fatigue damage models [8]. Depending on the scale level and the degree of detail needed, these models are divided into the following categories: fatigue life models (e.g., [9,10]), phenomenological models (e.g., [11,12]), and progressive damage models (e.g., [13,14]). However, in comparison to the high number of valid static failure models for FRP (e.g., [15,16]), there exist no general valid model for the fatigue life prediction of these materials.

In [17,18] a fatigue damage model (FDM) has been proposed for the meso- and macro-scale level which combines the advantages of the various models’ classes. This FDM is layer-based and physically motivated. Furthermore, the basic version of the model has the potential to analyze load sequence effects and to consider load redistributions under pulsating loading with constant stress ratio. Recently, the model has been extended in [19] to consider three-dimensional stress states in FRP as well as to analyze hybrid laminates [20].

### 1.2. Goals and Overview of the Present Article

In extension of this scientific work, the damage phenomenology regarding fatigue loads with load reversals on different length scales is investigated and mathematically described in this collaborative study. The mesoscopic damage initiation and progression in cross-ply laminates is analyzed for describing the material degradation during fatigue and micromechanical explanations for the observed behavior are worked out using neat resin experiments and representative volume element (RVE) simulations for constant and fatigue loading.

The main objectives of this paper are: (1) The experimental investigation of the damage phenomenology under cyclic loading with load reversal on different observation scales, (2) the analysis and description of micromechanical damage by means of microscale experiments and finite element modeling using an RVE, (3) linkage of the micromechanical findings with the mesoscale damage evolution and elaboration of the influence of constant amplitude and block loadings and (4) the development of numerical methods to consider the effects of load reversals and load sequence on fatigue damage evolution.

**Figure 1:**
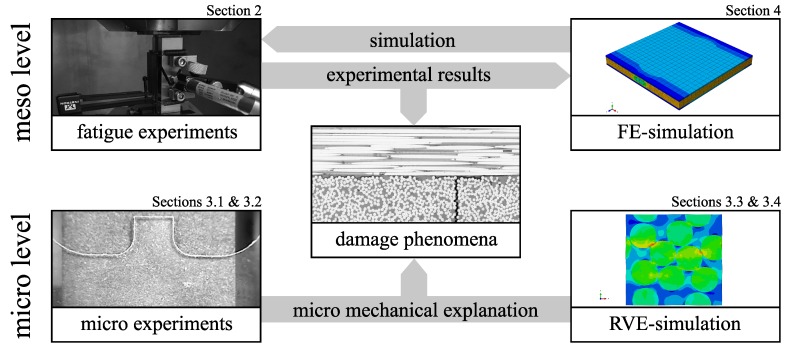
Overall structure of the paper and connections between the individual sections.

The present research therefore analyses the fatigue behavior of CFRP at the meso and micro scale in order to elaborate the driving mechanisms of damage initiation and growth under different load ratios in Section 2 and Section 3. Furthermore, micromechanical FE-analyses are carried out in Section 3 to investigate the stress-strain states within the off-axis layer. Conclusively, an attempt is made to extend an existing FE model with respect to the experimental findings in Section 4. An overview of the work and its individual assignment to the corresponding sections is given in Figure 1.

## 2. Damage Behavior on the Layer Level

### 2.1. Materials and Experimental Methods

The specimens used within this study were manufactured from a T700SC carbon fiber and an Araldite LY556 epoxy resin system. The unidirectional (UD, $\left[0_{11}\right]$) and bidirectional (BD, $\left[0_{2}/90_{7}/0_{2}\right]$) lay-ups were made by dry filament winding and subsequent resin transfer molding (RTM). After curing for 3 h at 80 ${}^{\circ }$C and post-curing at 150 ${}^{\circ }$C for 4 h, the specimens were cut out of the laminated plates by water jet (UD-specimens), or abrasive cutting machine (BD-specimens). The cross-ply specimens were applied with aluminum end-tabs and polished at the edges with a diamond suspension (minimal grain size 1 μm) to ensure clear visibility of the microstructure. Full details on the characterization process and the experimental procedure are given in [7]. The material properties obtained from the UD-specimens are summarized in Table 1.

Additionally, bulk epoxy specimens were prepared for the micromechanical tests using the RTM process and a geometry derived from the DIN EN ISO 527-2 standard. The final bulk specimen geometry was then established using an CNC-milling machine.

**Figure 2:**
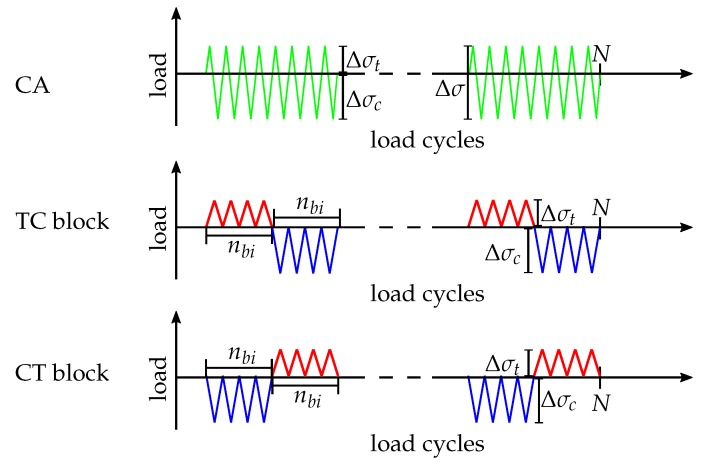
Schematic illustration of the loading sequences, constant amplitude loading (CA), tension-compression (TC) and compression-tension (CT) sequence. Block lengths within one experiment are equal for tension and compression blocks. Two different block lengths $n_{bi}$ are under investigation and tested in distinct experiments.

Fatigue tests with load reversals were performed for $n=5\times 10^{5}$ cycles at a frequency of f=6 Hz with a stress ratio of R=−3.26 and a maximum applied stress of $\sigma_{max}^{lam}=105$ MPa. An anti-buckling device was used in all the tests to avoid specimen buckling under compression. Block-loading experiments with repeated block lengths $n_{b1}=5\times 10^{3}$ cycles and $n_{b2}=5\times 10^{4}$ cycles were conducted until a total number of cycles of $n=5\times 10^{5}$ in tension-compression (TC) and compression-tension (CT), respectively. Block lengths were kept constant within the individual experiments. The amplitudes and mean stresses of the tension and compression blocks correspond to the tension ($\Delta \sigma_{t}$) and compression ($\Delta \sigma_{c}$) fractions of a cycle with R=−3.26 and a total stress range Δσ, hence in tension $\sigma_{min}=0$ MPa and in compression $\sigma_{max}=0$ MPa. The block lengths in tension and compression are equal within one experiment, hence at the end of a fatigue test the same amount of tension and compression cycles have been applied to the specimen. A clarifying illustration is given in Figure 2. Consequently, the influence of different block lengths as well as the effect of the tension and compression sequence is addressed. Crack densities and delamination lengths are recorded regularly by microscopy. Therefore, the specimens are removed from the testing machine and examined without any external loading.

The results of the fatigue experiments deliver insight into the damaging process of the CFRP-laminate helping to understand the behavior on the macro-level, e.g., in terms of stiffness loss. However, the damage phenomena itself occur on the meso and micro-level. Therefore, the damage phenomena are investigated here in detail and will serve to link the determined cracking process in the case of manifold loading conditions with the microscopic mechanisms within the matrix and the fiber-matrix-interphase.

### 2.2. Damage Evolution in Cyclic Loading

**Figure 3:**
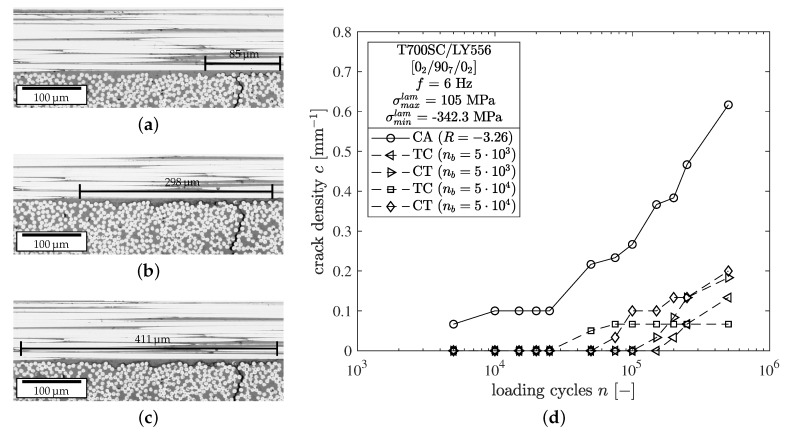
Consecutive edge micrographs of a delamination growing from a microcrack at $\sigma_{x}^{lam}=$ 105 MPa with R=−3.26 after (**a**) n=5000, (**b**) n=15 as well as (**c**) n=25,000 and (**d**) crack density evolution for constant amplitude (CA) and block-loading (TC/CT) experiments.

In the following, the experimental results on the layer level are summarized for the CA, TC, and CT tests with respect to the observed crack and delamination growth. Therefore, the damage is quantified in terms of the crack density $c=\sum_{i}^{m}k_{i}/2l_{0}$ of the 90${}^{\circ }$-layer and the lengths of the delaminations *a* emanating from the crack tips. The number of microcracks $k_{i}$ perpendicular to the loading direction is determined at both polished edges of the specimens regularly within an observation length $l_{0}=30$ mm. The specimens were unmounted from the testing machine to obtain the micrographs. Simultaneously, the lengths of the crack tip delaminations were measured for every crack at one specimen edge. The delamination lengths are determined from tip to tip from the micrographic images. As seen from Figure 3a,c, the delamination is clearly visible at the specimen edge and is determined regularly throughout the cyclic experiment.

**Figure 4:**
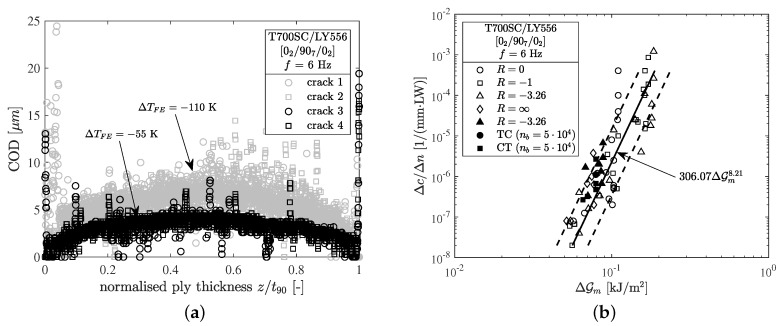
Comparison of the (**a**) residual CODs of the specimens tested previously (light symbols) [5,6] and specimens tested at present (dark symbols) as well as (**b**) crack density growth rate Δc/Δn vs. the range of the strain energy release rate $\Delta G_{m}$ for the initiation of a microcrack.

In terms of crack density evolution only minor crack growth up to *c* ≤ 0.2 mm${}^{-1}$ was observed in the block-loading experiments after $n=5\times 10^{5}$ cycles, regardless of the block lengths. However, block-loading experiments with a CT sequence tend to show slightly higher crack densities compared to a TC sequence. In contrast, the fully reversed experiment without block-loading shows a significantly pronounced crack growth up to c=0.616 mm${}^{-1}$ at the end of the test. The results of the crack countings are shown in Figure 3d. As seen from Figure 4b the measured crack growth rates are comparably low throughout the whole experiment. The depicted Paris-relation $\Delta c/\Delta n=306.07\Delta G_{m}^{8.21}$ and scatter band were determined from the previous experiments (hollow symbols). It can be seen that the results of the block-loading experiments fit well into the existing scatter band.

Analysis results in terms of delamination growth show similar trends. No remarkable difference between the block-loading experiments has been observed, either in terms of block length or in terms of block sequence. It can be stated once more that the fully reversed test without block-loading shows more pronounced delamination initiation and growth, meaning that more and larger delaminations have been determined.

After the first n=5000 cycles high resolution micrographs with 500-fold magnification were taken to compare the residual crack opening displacement (COD) with the results from experiments published earlier by Koch et al. [5,6]. In both cases the specimens were examined after the first n=5000 loading cycles. Cracks 3 and 4 represent the present specimens revealing a significant reduction of the residual crack opening displacement compared to the former set of specimens from the same manufacturing batch, as seen from Figure 4a. The corresponding temperature differences that invoke the same COD calculated by finite element method (FEM) are $\Delta T_{FE}=-110$ K and $\Delta T_{FE}=-55$ K for the former and present specimens, respectively. In terms of residual stresses this means a reduction from $\sigma_{22}^{90,res}=25.7$ MPa to 15.3 MPa, hence slower crack growth has to be expected when testing at the same maximum loads.

### 2.3. Interpretation of Results

As already pointed out in [5,6], residual stresses have a significant influence on the damaging behavior of cross-ply laminates. Therefore, an attempt is made to explain the observed experimental phenomena with respect to the local stress state within the 90${}^{\circ }$-layer.

**Figure 5:**
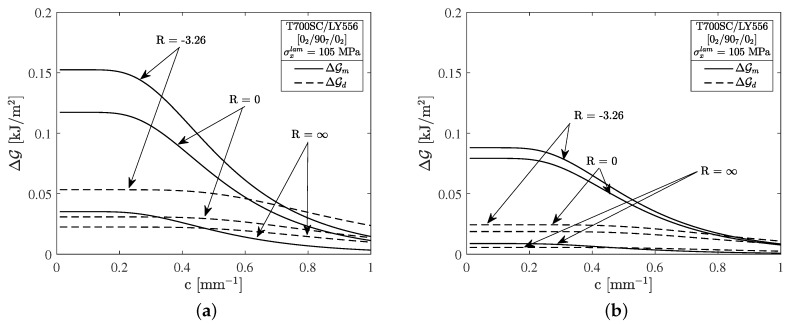
The strain energy release rate for the formation of a microcrack $G_{m}$ compared to the energy release rate for the initiation of a crack tip delamination $G_{d}$ for different load ratios and residual stresses within the 90${}^{\circ }$-layer of magnitudes (**a**) $\sigma_{22}^{90,res}=25.7$ MPa and (**b**) $\sigma_{22}^{90,res}=15.3$ MPa, respectively.

Residual stresses can be understood as an additional static load that is added to the applied cyclic loading. In consequence, the 90${}^{\circ }$-layer is subjected to higher maximum stresses and the mean stress is shifted to higher values, implying a higher local stress ratio of the embedded 90${}^{\circ }$-layer. The residual stresses also contribute a significant base amount of strain energy in terms of crack formation, hence a reduction of residual stresses will lead to delayed crack formation in static as well as fatigue loading. Figure 5a,b emphasize the influence of the residual stresses on the energy release rate (ERR) for crack formation and delamination initiation. The strain energies shown here were calculated by means of the variational model from Nairn as proposed in [21].

Figure 5a shows the crack density dependent ERR for a high amount of residual stresses ($\sigma_{22}^{90,res}=25.7$ MPa) and an applied laminate stress of $\sigma_{x}^{lam}=105$ MPa. Accordingly, loadings with R=−3.26 lead to the highest amount of strain energy release rate. The ERRs for R=0 and R=∞, which correspond to tension and compression block-loading, respectively, are significantly lower. This also holds in terms of strain energy for delamination growth $\Delta G_{d}$, as depicted by the dashed lines in Figure 5a. In the case of R=∞ it is assumed that only the residual stresses contribute to the ERR. This is argued with respect to the observed fracture modes of the cracks, which have been found to be perpendicular to the loading direction. It is further argued that compression stresses have no damaging effect.

The influence of residual stresses is further highlighted by Figure 5b, which depicts the ERRs for the same maximum tension stress $\sigma_{x}^{lam}=105$ MPa but reduced residual stresses $\sigma_{22}^{90,res}=15.3$ MPa complying with the results from the COD measurements from Figure 4a. The ERR for R=−3.26 is approx. halved and significantly lowered in cases R=0 and R=∞ compared to the previously conducted experiments.

As depicted in Figure 4b, a comprehensive crack growth relation comprising several load ratios can be successfully established by calculating the ERRs for the experiments with respect to the residual stresses and using the range of the ERR $\Delta G_{m}$ as the driving force for microcrack formation in cross-ply laminates. It can be seen that the results for the different load ratios, constant amplitude and block-loading experiments as well as specimens with different residual stress states collapse to a single scatter band. In the authors’ opinion this crack growth relation is characteristic for the tested material and can be used for calculating the crack growth of cross-ply laminates at different load ratios and varying maximum stresses.

## 3. Micromechanical Explanations for the Influence of Load Reversals

In the previous section the results of block-loading experiments with TC-, CT sequences as well as constant amplitude loading is presented in terms of the crack evolution and the influence of residual stress. To understand the underlying mechanisms of the crack formation in matrix, investigations on the micromechanical level were carried out. Based on experiments and numerical studies, Hopmann et al. [22] have shown that the viscoelasticity of the matrix remarkably determines the stress distribution in UD after long-term loading and can thus influences the fatigue life of the composites. Therefore, investigation with resin creep and fatigue experiments was conducted (cf. Section 3.1 and Section 3.2) and micromechanical simulation was carried out, which takes the fiber distribution and the viscoelasticity of the matrix into consideration (cf. Section 3.3, Section 3.4 and Section 3.5).

### 3.1. Residual Mechanical Properties after Creep and Fatigue Loading

To understand the influence of the neat resin on the macroscopic damage initiation process microscopic samples (0.5 mm × 5 mm cross section) were tested. Using these results a connection between the observed microscopic effects and the detected macroscopic crack density variation shall be established.

**Figure 6:**
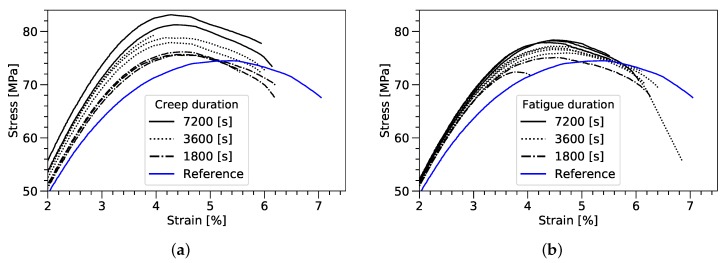
Tensile tests showing the residual strength after creep (**a**) and fatigue loading (**b**), a stress of 43 MPa at 50${}^{\circ }$ has been used.

The samples were loaded in tension-tension fatigue (amplitude = 4.3 MPa; mean stress = 43 MPa) to exemplary investigate the behavior of the epoxy resin in the transversely loaded plies. The observed residual stress after manufacturing and static loads were simulated by tensile-creep loading. Three loading durations were chosen between 1800 s and 7200 s applying a mean static stress of 43 MPa and a temperature of 50${}^{\circ }$, respectively. After unloading to a pre-load of 2 N the specimens were immediately tested in a quasi-static tensile test. The results are shown in Figure 6. Generally, in contrast to a decreasing stiffness on the macroscopic level, the study shows an increase of strength and stiffness of the neat resin samples in the quasi-static test along with a reduction of the fracture strain.

The ultimate tensile strength increases for both test configurations in comparison to the reference sample, where an average ultimate tensile strength of 74.5 MPa has been measured. The effect of increasing strength during the tensile test can be consistently observed with increasing load duration for fatigue, as well as, creep loading. While the creep loading shows an increase of up to 11% (83.1 MPa) the fatigue samples show a lower strength gain of 2.3% (76.2 MPa) after 3600 s and 5.2% (78.4 MPa) after 7200 s load duration. In both test cases the reduction of the fracture strain can be related to the creep and fatigue induced strain variation.

The origin of this effect is, beside others, assumed to relate to the orientation of epoxy polymer chains as observed by Scherzer [23]. Further studies must be performed to fully understand the contribution of creep and cyclic loads on the micromechanical level towards macroscopic damage initiation.

### 3.2. Transferability of Micromechanical Matrix Observations on Mesoscopic Level

Considering the observed increasing stiffness and strength of the matrix due to long-term static and fatigue tension loading, in the following possible effects on the fatigue behavior of the unidirectional fiber-reinforced layer are discussed. For that it is assumed that the polymer matrix near the fiber as well as the neat resin regions behave in a comparable way in the composite, keeping in mind that Fiedler et al. [24] have previously shown that the neat resin may behave differently from bulk epoxy.

Beside others, two complex interacting effects of the observed stiffening and strengthening of the matrix are pointed out: matrix stiffening may lead to stress redistributions to the detriment of the interphase and matrix strengthening to reduction of matrix failure index. Depending on the material composition, meaning the stiffness ratio of matrix and fiber, condition of the fiber-matrix interphase, fiber volume fraction etc. a positive or negative cumulative effect of tension prestresses (e.g., at 0<R<1) on the damage behavior of fiber-reinforced composites is expected. As an example, a positive effect is expected for the case of a well-conditioned interphase with strength reserves. Here stress redistributions towards the interphase and matrix strengthening superpose positively. A contrary effect is expected for comparable weak interphases.

In the next section it will be shown that viscoelastic effects of that type diminish under reversed constant amplitude and block-loading. For the given material and its specific material composition these effects contribute to the mesoscopic damage behavior as observed and modeled analytically in Section 2.2. In the future further scientific effort will be made for understanding the contribution of local material phenomena to the mesoscopic and macroscopic damage behavior.

### 3.3. Micromechanical Modeling of the UD-Layers

Besides the changed stiffness and residual strength, the viscoelasticity of the resin also directly influences the stress distribution within composite materials. Based on experiments on UD-specimens and numerical studies, Hopmann et al. [22] have previously shown that the viscoelasticity of the matrix remarkably determines the micromechanical stress and strain redistribution after creep and fatigue loading and influences the fatigue life of the composites.

To analyze the micromechanical stress and strain state in the 90${}^{\circ }$-layers of a $\left[0_{2}/90_{7}/0_{2}\right]$ laminate during the fatigue tests, numerical investigations with three-dimensional RVE representing the 90${}^{\circ }$-layers were carried out. The focus of the study is the stress and strain condition in 90${}^{\circ }$-layers, the 0${}^{\circ }$-layers were not explicitly modeled. Instead, the interaction between the 90${}^{\circ }$- and 0${}^{\circ }$-layers during manufacturing and mechanical tests was described by equivalent boundary conditions of the RVE (cf. Section 3.4).

For the 90${}^{\circ }$-layers under transverse loading, the local fiber arrangement significantly affects the stress and strain distribution in the matrix. Therefore, the fiber distribution was considered in the modeling with RVE. For generating quasi-random fiber distributions, the *Random Sequential Expansion* (RSE) algorithm from Yang et al. [25] was implemented, which adds new fibers sequentially around existing fibers and enables a higher fiber volume fraction above 60%. With this algorithm, an RVE-model with 24 fibers and a fiber volume fraction of 61% was generated. The fibers intercepted by the model boundaries were divided and the complementary parts were positioned along the opposite boundaries to ensure geometric periodicity of fiber arrangement.

For the fiber region, coupled temperature-displacement elements with reduced time integration (C3D8RT) were used and for the matrix, coupled temperature-displacement elements (C3D8T) were used. To ensure a periodic deformation of opposite boundaries and zero averaged stress gradients over the model, the following boundary conditions were defined for the nodes on model boundaries:

(1) $$\begin{array}{cc} \vec{u_{i}}\left(x,y,z\right)-u_{i}^{p}\left(x,y,z\right) & =\varepsilon_{ij}^{0}\;\cdot \;l_{i},\\ \vec{u_{i}}\left(0,y,z\right)-{\vec{u}}_{i}\left(l_{x},y,z\right) & =\varepsilon_{ix}^{0}\;\cdot \;l_{x}\;\forall \Gamma_{x},\\ \vec{u_{i}}\left(x,0,z\right)-{\vec{u}}_{i}\left(x,l_{y},z\right) & =\varepsilon_{iy}^{0}\;\cdot \;l_{y}\;\forall \Gamma_{y},\\ \vec{u_{i}}\left(x,y,0\right)-{\vec{u}}_{i}\left(x,y,l_{z}\right) & =\varepsilon_{iz}^{0}\;\cdot \;l_{z}\;\forall \Gamma_{z}, \end{array}$$

where $u_{i}$ represents the displacement of the nodes on boundaries Γ in *i*-th direction, $\varepsilon_{ij}$ is the global strain of the model and *l* is the length of the model in *i*-th direction. This boundary condition ensures the periodicity of the modeled section for representing the entire UD-layer under loading [26].

The material behavior of the fibers was assumed to be transversal isotropic and linear elastic. The transverse elastic properties of the T700SC fibers were assumed to be similar to T300 fiber and the values were taken from [27].

Based on the experimental observations in [22], the resin system was modeled as non-linear viscoelastic using a material model developed by Brandt and Küsters [28,29]. The model was implemented as a user-defined material subroutine (UMAT) for the finite element software ABAQUS. The model assumes that the volumetric deformation is linear elastic, i.e., the bulk modulus *K* is constant. The time dependence is only determined by the deviatoric component. The viscoelasticity is described by a generalized Maxwell-model with a mathematical formulation of Prony series, Equation (2), in which the stiffness parameters $G_{\infty }$, $G_{i}$ and the relaxation times $\tau_{i}$ are to be determined experimentally.

(2) $$\begin{array}{c} G\left(t\right)=G_{\infty }+\sum_{i=1}^{20}G_{i}\exp\left(-t/\tau_{i}\right) \end{array}$$

The model takes the influence of stress level and temperature into consideration. The stress-dependent Prony-coefficients, e.g., the relaxation time $\tau_{i}$ of each of the 20 Maxwell-elements are calibrated and described in a table for nine deviatoric stress levels (10–90 MPa). The time/temperature-shifting principle models the influence of temperature. The temperature-dependent shifting factors $a_{T}$ are modeled with the modified van’t Hoff shifting approach [29] shown in Equation (3):

(3) $$\begin{array}{c} ln\left(a_{T}\right)=-B/\sqrt{C}\left[\arctan\left(\sqrt{C}\left(T-T_{m}\right)\right)-\arctan\left(\sqrt{C}\left(T_{0}-T_{m}\right)\right)\right], \end{array}$$

where $T_{0}$ represents the reference temperature and the parameters *B*, *C* and $T_{m}$ are to be determined experimentally.

Küsters [29] has discussed different approaches to determine the Prony-coefficients and the parameters of the modified van’t Hoff equation. In this study, strain-controlled unidirectional tension tests were performed on bulk epoxy specimens at three temperatures (40${}^{\circ }$, 60${}^{\circ }$ and 80${}^{\circ }$) and three strain rates (120 %/h, 1200 %/h and 12,000 %/h). Stress/strain curves were measured for each combination of temperature and strain rate.

**Figure 7:**
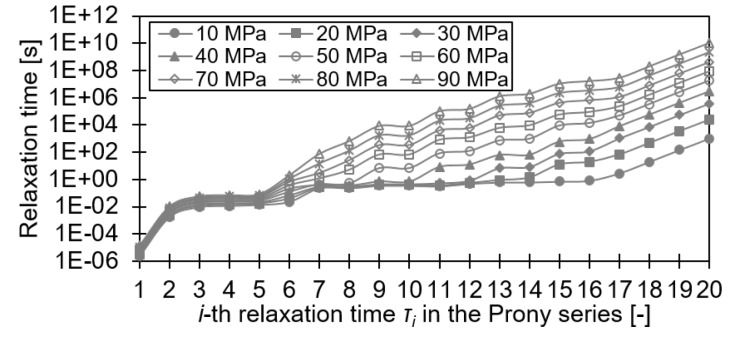
Stress-dependent relaxation times of the Prony series in Equation (2).

Based on these measurements, the Prony-coefficients for each stress level as well as the parameters of the modified van’t Hoff equation were calibrated. A solver implemented by Küsters [29] was used to iteratively determine the Prony-coefficients by minimizing the total error when reproducing the stress/strain curves. The parameters obtained are summarized in Table 2 and Figure 7.

**Figure 8:**
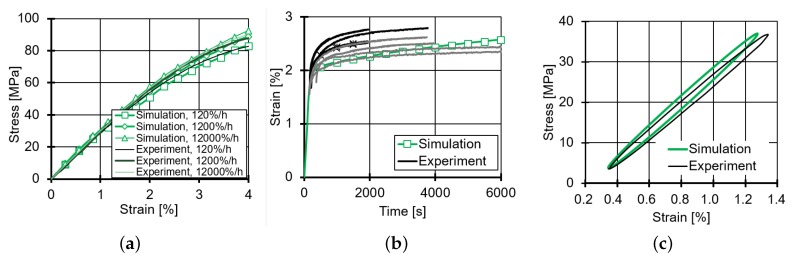
(**a**) Stress-strain relation of bulk resin in tension tests under different strain rates at 23${}^{\circ }$; (**b**) progress of strain of bulk resin under creep loading with 43 MPa at 50${}^{\circ }$; (**c**) stress-strain relation of a UD-layer under cyclic longitudinal shear loading with R=0.1 at 25${}^{\circ }$.

The simulation results with the RVE-model and the material model for the matrix is compared with experimental results for different load cases (cf. Figure 8). The simulation is in good agreement with the experimental results. In the case of creep loading (cf. Figure 8b), the simulated curve of bulk epoxy is in the scatter band of the experiment. In particular, the creep strain increment in the first 2000 s can be very well predicted, which corresponds to the duration of the first 12,000 cycles in the fatigue tests. The RVE-simulation results are also in accordance with the hysteresis of a UD-layer under shear loading (cf. Figure 8c). The overestimation of dynamic shear stiffness might be a consequence of the assumed transverse stiffness of T700SC fibers. Thus, the modeling with the RVE and the matrix model is verified and validated. In the following, the micromechanical stress and strain state in the matrix during the fatigue tests will be analyzed with this model and discussed.

### 3.4. Modeling Setup for the Simulation of Micromechanical Stress and Strain States in Cyclic Tests

The loading condition of the 90${}^{\circ }$-layer in a $\left[0_{2}/90_{7}/0_{2}\right]$ laminate was described by equivalent boundary conditions. In the first load step, the thermal manufacturing influence was considered by imposing the post-curing temperature profile and a storage time of 7 days at room temperature onto the model. In the laminate coordinate system, the global displacement of the RVE in 0${}^{\circ }$-direction was kept to zero to model the strain obstruction by the 0${}^{\circ }$-layers. Subsequently, each loading block (compression, tension or fully reversed loading) was modeled with a strain-controlled cycle-jump technique. The strain of the 90${}^{\circ }$ layer $\varepsilon^{RVE}$ was calculated as $\sigma^{lam}/E^{lam}$. The first and the last load cycles in each block were explicitly modeled between $\varepsilon_{max}^{RVE}$ and $\varepsilon_{min}^{RVE}$ with 6 Hz. The cycles in between were replaced by a mean strain relaxation step to consider the viscoelastic stress redistribution in the matrix while keeping a low computational effort.

### 3.5. Influence of Loading Sequence on the Stress and Strain State

For the CT and TC block-loading, two blocks were simulated, respectively. For the CA load case, 5000 cycles were simulated. The strain and stress of every integration point in the matrix region after the tension, compression, or CA blocks will be analyzed hereafter. Moreover, the span of octahedral matrix stress within one loading cycle will also be compared for the different loading cases. The span of the octahedral shear stress in the principle stress space within a loading cycle is defined as:

(4) $$\begin{array}{c} \begin{array}{cc} \tau_{oct} & =\frac{1}{3}\sqrt{{\left(\sigma_{1}-\sigma_{2}\right)}^{2}+{\left(\sigma_{2}-\sigma_{3}\right)}^{2}+{\left(\sigma_{1}-\sigma_{3}\right)}^{2}},\\ \Delta \tau_{oct} & =\tau_{oct,max}-\tau_{oct,min}. \end{array} \end{array}$$

For the span of the octahedral normal stress, only the positive section of the oscillating normal stress is calculated. This is based on the assumption that the tensile stress is more critical than compressive stress in terms of crack initiation. Since no cracks were explicitly modeled, the stress spans indicate the possibility of crack nucleation for different matrix locations.

(5) $$\begin{array}{c} \begin{array}{cc} \sigma_{oct} & =\frac{1}{3}\left(\sigma_{1}+\sigma_{2}+\sigma_{3}\right),\\ |\Delta \sigma_{oct}^{+}| & =|H\left(\sigma_{oct,max}\right)\sigma_{oct,max}-H\left(\sigma_{oct,min}\right)\sigma_{oct,min}|, \end{array} \end{array}$$

where *H* is the Heaviside function which is defined as:

(6) $$\begin{array}{c} \begin{array}{c} H(\sigma )=1\;\mathrm{for}\;\sigma \geq 0,\\ H(\sigma )=0\;\mathrm{for}\;\sigma <0. \end{array} \end{array}$$

**Figure 9:**
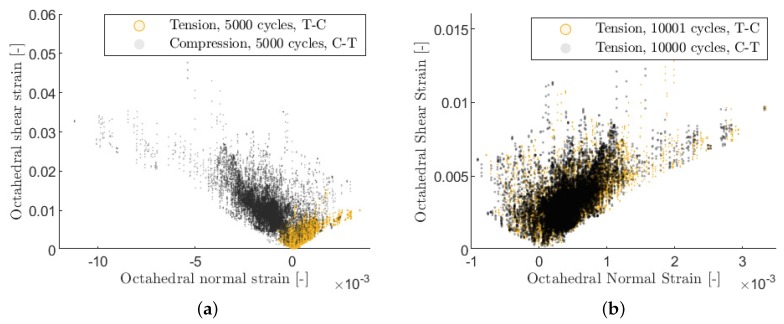
(**a**) Comparison of the strain distribution in matrix after one tensile or compressive block in T-C and C-T sequence (**b**) comparison of the strain distribution in matrix after two tension and compression blocks in T-C and C-T sequence.

Firstly, the strain distributions in the matrix in TC and CT block-loading are compared. A clear difference in the strain distribution after a tension or a compression block can be observed (cf. Figure 9a). Generally, the compression block leads to lower octahedral tensile strain but higher shear strain in the matrix. However, after a full TC or CT sequence, i.e., after the 90${}^{\circ }$-layer undergoes the same amount of tension and compression cycles, the strain distribution in both cases is only marginally different (cf. Figure 9b). A very similar strain distribution was also observed under fully reversed loading (CA) after the layer undergoes the same amount of tension and compression cycles (thus not shown). From a continuum mechanics point of view, this indicates that different loading sequences with same stress levels have no significant influence on the strain distribution in matrix under the given assumptions.

**Figure 10:**
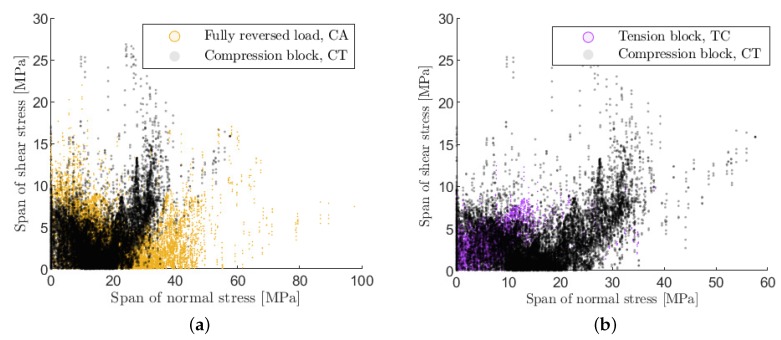
(**a**) Comparison of the stress span within a fully reversed cycle (CA) and within a compression block (CT) (**b**) comparison of the stress span within a compression block (CT) and within a tension block (TC).

Figure 10 shows however a clear difference between the stress span within a fully reversed cycle (CA) and a tension or compression cycle under TC or CT loading. This can be explained by the residual stresses, which generally shifts the initial stress distribution in matrix into tension. In a fully reversed cycle, this tensile residual stress is further increased in the tension phase and fully compensated in the compression phase. Therefore, the positive stress span in a fully reversed cycle is higher than in a tension or compression cycle under TC or CT loading. The difference in the stress span is in accordance with the different ERRs on the layer level (see Section 2.3) and explains the faster crack density evolution observed in fully reversed experiments from a continuum mechanical point of view.

## 4. FE-Based Damage Modeling

The micromechanical analyses in Section 3 has been carried out to address the influence of creep at mean stress and stress redistribution due to fatigue loadings. Together with the experimental results from laminate experiments in Section 2 the dependence of the crack density growth in the 90${}^{\circ }$-layer on the applied stress amplitude and the maximum stress, the residual stresses and the mean stress is shown. Macroscopically the crack density is directly related to the stiffness loss of FRP, as a high number of cracks relates to a high stiffness loss. The experimental findings are hereafter incorporated into an existing FDM [17]. The FDM is extended to account for the influence of residual stresses and load reversals. Details on the general model approach and the implemented extensions are provided.

### 4.1. An Energy-Based Fatigue Damage Model for Analyzing Load Reversals

#### 4.1.1. Basic Operation of the FDM

The originally developed FDM by Krüger [17,18] is based on classical lamination theory and has been implemented in the commercial finite element software ABAQUS as a user-defined material subroutine (UMAT) for layered-shell elements. Based on the applied loads (e.g., tension, compression and shear) and the material orientations *j* (e.g., longitudinal and transverse to the fiber direction) strength and stiffness degradation factors ($\eta_{R_{j}}$ and $\eta_{E_{j}}$, respectively) are introduced in order to describe the damage state ($\eta_{R_{j},E_{j}}=0$ completely damaged; $\eta_{R_{j},E_{j}}=1$ undamaged):

(7) $$\begin{array}{c} \begin{array}{cc} E_{j,da} & =\eta_{E_{j}}\;\cdot \;E_{j},\\ R_{j,da} & =\eta_{R_{j}}\;\cdot \;R_{j}. \end{array} \end{array}$$

The FDM comprises of two main parts, a discontinuous and a continuous part. In the discontinuous part, the degradation is analyzed under static loading using Puck’s criterion [15] for static failure. In the continuous part, the degradation is calculated due to cyclic loading [18].

The main feature of the FDM is the energy-based hypothesis of Pfanner [30]. This hypothesis implies that the damage state, represented by strength and stiffness degradation, of a quasi-statically loaded material is comparable with that of a cyclically loaded material, if the magnitude of energy dissipated in damage is equal ($g^{st}=g^{fat}$).

**Figure 11:**
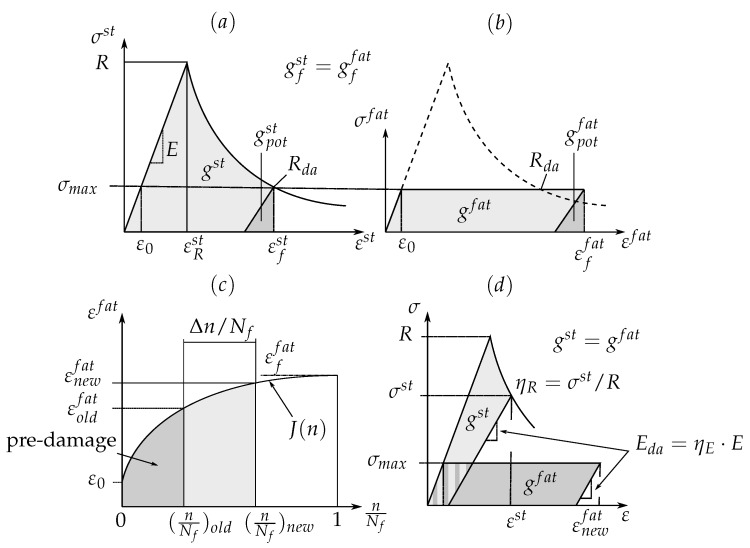
Schematic illustration of the determination of the stiffness ($\eta_{E}$) and strength ($\eta_{R}$) degradation factors: (**a**) static stress-strain curve; (**b**) virtual cyclic stress-strain curve; (**c**) strain/damage evolution depending on normalized load cycles; (**d**) determination of the degradation factors $\eta_{E}$ and $\eta_{R}$, see [18].

The damage calculation procedure is explained using, as an example, an embedded $90^{\circ }$-layer under transverse tension loading, see Figure 11. To apply Pfanner’s hypothesis, pre- and post-critical stress-strain curves have to be available. Firstly, with the help of the computed maximum stresses $\sigma_{max}$ and the corresponding quasi-static stress-strain curves, the amount of dissipated energy until failure $g_{f}^{st}$ are obtained, see Figure 11a. Subsequently, after the determination of the dissipated energy $g_{f}^{st}$, the application of the energy hypothesis ($g_{f}^{st}=g_{f}^{fat}$) yields the values of the failure fatigue strains $\varepsilon_{f}^{fat}$, see Figure 11b. Next, depending on the initial strains $\varepsilon_{0}$ and the failure fatigue strains $\varepsilon_{f}^{fat}$, the damage evolution curves J(n) are defined, see Figure 11c. The characteristics of these curves refer to experimental observations. By means of the damage evolution curves J(n) the fatigue strains $\varepsilon^{fat}$ are obtained depending on the normalized life $n/N_{f}$ for the current calculation step. The normalized life $n/N_{f}$ is depicted based on the given number of load cycles *n* for a certain load block and the number of load cycles to failure $N_{f}$. The values of $N_{f}$ are determined from *S*-*N* curves. If the material is pre-damaged, the pre-damage is converted in the form of dissipated energy to the stresses $\sigma_{max}$ at the current loading block, depending on the fatigue strains $\varepsilon^{fat}$ and the normalized life $n/N_{f}$. Therefore, regardless of how the pre-damage has been developed, only the amount of dissipated energy matters. With increasing number of load cycles *n*, the fatigue strains $\varepsilon^{fat}$ and the dissipated energy increase, and therefore the damage evolves. The updated degradation factors for strength and stiffness are then determined iteratively also with the help of the stress-strain relationship and the Pfanner hypothesis [30], see Figure 11d. Fatigue failures occur when the residual strengths $R_{da}$ are degraded to the corresponding applied stress levels $\sigma_{max}$. A detailed description of the original FDM can be found in [17].

#### 4.1.2. Extension of the FDM

In a first step, the FDM was calibrated for the selected material T700SC/LY556 based on the material characterization given in Section 2.1. With the help of the stress-strain relationships resulting from the quasi-static tests, the static modeling approaches implemented in the FDM as well as the failure criterion according to Puck could be verified. A description of the FDM calibration procedure for T700SC/LY556 is provided in [31].

To use the FDM for the analysis of variable load amplitude cases, additional modeling elements are required. One of the main ingredients in the damage calculation in such cases is the S-N curves, which depend on, among others, on the applied stress ratio and the fiber orientation. Based on [32], Constant Fatigue Life Diagrams (CFL) were constructed and implemented in the FDM [31] to create different S-N curves. By using the CFL, the number of cycles to failure given by the S-N curves can be determined for any stress ratio, which is an important parameter for the damage evolution relationships contained in the FDM [18,31]. The procedure for determining the different S-N curves is described in detail in [31].

**Figure 12:**
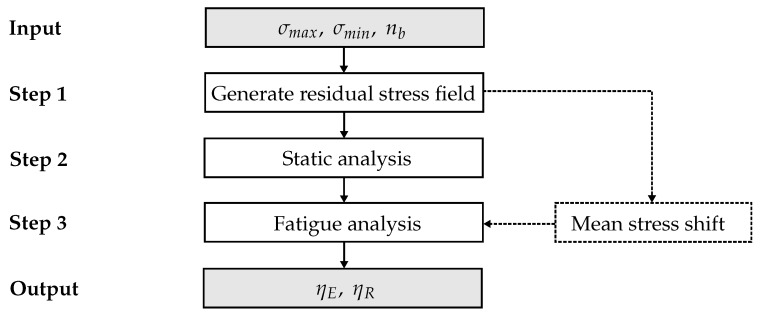
Extended simulation framework.

In a further step towards a realistic fatigue analysis, the quantified effects of the manufacturing-induced residual stresses as shown in Section 2.2 are taken into account. As described in Section 2.3, they significantly influence the damage behavior in the embedded $90^{\circ }$-layer. In the simulation framework residual stresses are considered to be follows. The residual stress field is generated prior to the fatigue simulation using a cooling simulation with the experimentally determined thermal expansion coefficients. Details of this procedure can be found in [33]. With the aid of the constructed CFL, the local change in mean stress or stress ratio, respectively, in the individual layers caused by the residual stresses is considered. To the repetition: The change of the stress ratios has a direct effect on the S-N curves and thus on the calculation of the number of load cycles until failure, which in turn is a specific value for the calculation of the fatigue damage evolution. Figure 12 shows schematically the extended computation procedure.

For a more realistic lifetime analysis, it is also necessary to consider crack closure effects [34] (passive damage) resulting from variable amplitude loading. Accordingly, in the FDM, the computation of the tension and compression stiffness degradation factors is to be coupled. It is assumed that the compression-induced damage influences subsequent tension-induced damage. This means that if compression damage is identified ($\eta_{E_{j}^{c}}<1$), the tensile stiffness degradation factors $\eta_{E_{j}^{t}}$ will also evolve under compression loading. On the other hand, it is assumed that the tension-induced damage has no influence on subsequent compression-induced damage:

(8) $$\begin{array}{c} \begin{array}{cc} \eta_{E_{j}^{t},new} & =\eta_{E_{j}^{t}}\;(\mathrm{update}\;\mathrm{at}\;\mathrm{tension}\;\mathrm{loading}),\\ \eta_{E_{j}^{c},new} & =\eta_{E_{j}^{c}}\;(\mathrm{update}\;\mathrm{at}\;\mathrm{compression}\;\mathrm{loading}),\\ \eta_{E_{j}^{t},new} & =\eta_{E_{j}^{t}}\;\cdot \;\eta_{E_{j}^{c}}\;(\mathrm{update}\;\mathrm{at}\;\mathrm{compression}\;\mathrm{loading}). \end{array} \end{array}$$

### 4.2. Application of the Simulation Framework for Load Scenarios with Load Reversals

**Figure 13:**
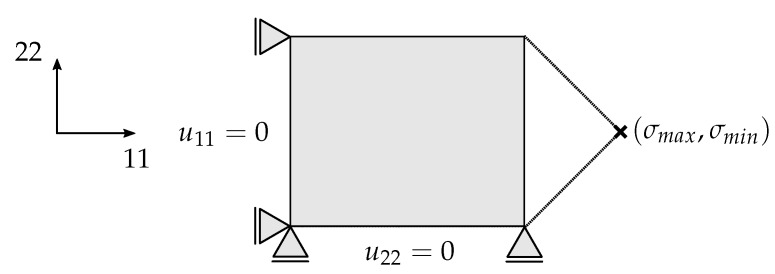
Mechanical model with boundary conditions for coupling to the FDM.

In the following, the results of a numerical study concerning the influence of load reversal on fatigue damage evolution using the extended simulation framework are presented. For the numerical study, the FDM is coupled with a full-integrated single layered-shell element (S4). This shell element is defined as a $\left[0_{2}/90_{7}/0_{2}\right]$ laminate with a nominal ply-thickness of $t^{ply}$ = 0.19 mm. For the numerical integration across the shell thickness, Simpson’s method is applied with three integration points in thickness direction per layer. In Figure 13, the schematic structure of the mechanical model including the associated boundary conditions is depicted.

The numerical simulations are performed under force-controlled loading. Furthermore, a block-wise analysis of the load cycles is carried out employing a cycle-jump technique to avoid the numerically inefficient cycle-by-cycle analysis. This block-loading strategy results from the applied energy approach according to Pfanner [30]. A detailed description of this procedure is described in [17]. For each load block, the maximum and minimum stress level, $\sigma_{max}^{lam}$ and $\sigma_{min}^{lam}$, respectively, as well as the number of load cycles per block $n_{b}$ are defined (cf. Figure 12).

As shown in Figure 12, in the first step of each damage analysis the manufacturing-induced residual stresses are determined. This is done by applying the thermal expansion coefficients ($\alpha_{\|}$, $\alpha_{⊥}$) listed in Table 1 with the aid of a negative temperature difference (cool-down) step as an initial state in the FE model. For the used $\left[0_{2}/90_{7}/0_{2}\right]$ laminate configuration, the predicted residual stresses are $\sigma_{11}^{90,res}=-15.33$ MPa and $\sigma_{22}^{90,res}=25.36$ MPa in the embedded 90${}^{\circ }$-layer. The calculated values are comparable with those determined from the COD analysis (see Section 2.2).

**Figure 14:**
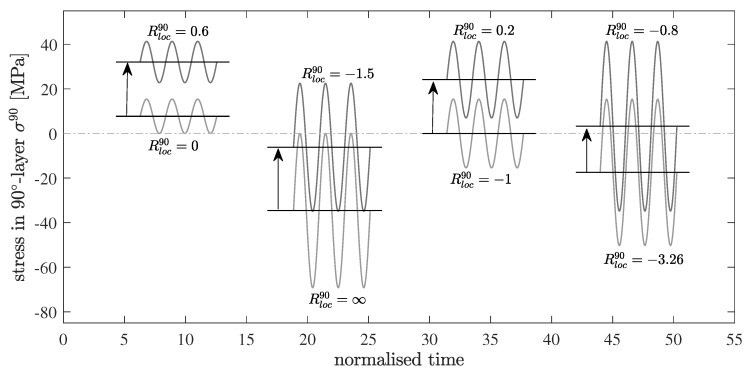
Schematic representation of the mean stress shift in the embedded $90^{\circ }$-layer caused by the manufacturing-induced residual stresses for different local stress ratios $R_{loc}^{90}$.

In the first part of this study, the effects of load reversals on the damage evolution at different stress ratios are investigated under constant amplitude loading (CA). Therefore, the following global stress ratios are selected: $R_{glob}^{lam}=0$ with $\sigma_{max}^{lam}=105$ MPa for a pulsating tension loading, $R_{glob}^{lam}=\infty$ with $\sigma_{min}^{lam}=-380$ MPa for a pulsating compression loading, and $R_{glob}^{lam}=\left\{-1,-3.26\right\}$ with $\sigma_{max}^{lam}=105$ MPa for alternating TC loading. Taking the thermal residual stresses into account, the local stress ratios $R_{loc}^{0,90}$ in the respective layers change. In the 90${}^{\circ }$-layer, there is, for example, a local alternating loading ($R_{loc}^{90}=-1.5$) at a global pulsating compression loading ($R_{glob}^{lam}=\infty$), whereas in a global pure alternating loading ($R_{glob}^{lam}=-1$) a local pulsating tensile loading ($R_{loc}^{90}=0.2$) was found. Global and local load ratios as well as maximum and minimum stresses within the 90${}^{\circ }$-layer are summarized in Table 3. In addition, Figure 14 schematically shows the changes of the mean stress for the different local stress ratios in the embedded $90^{\circ }$-layer.

**Figure 15:**
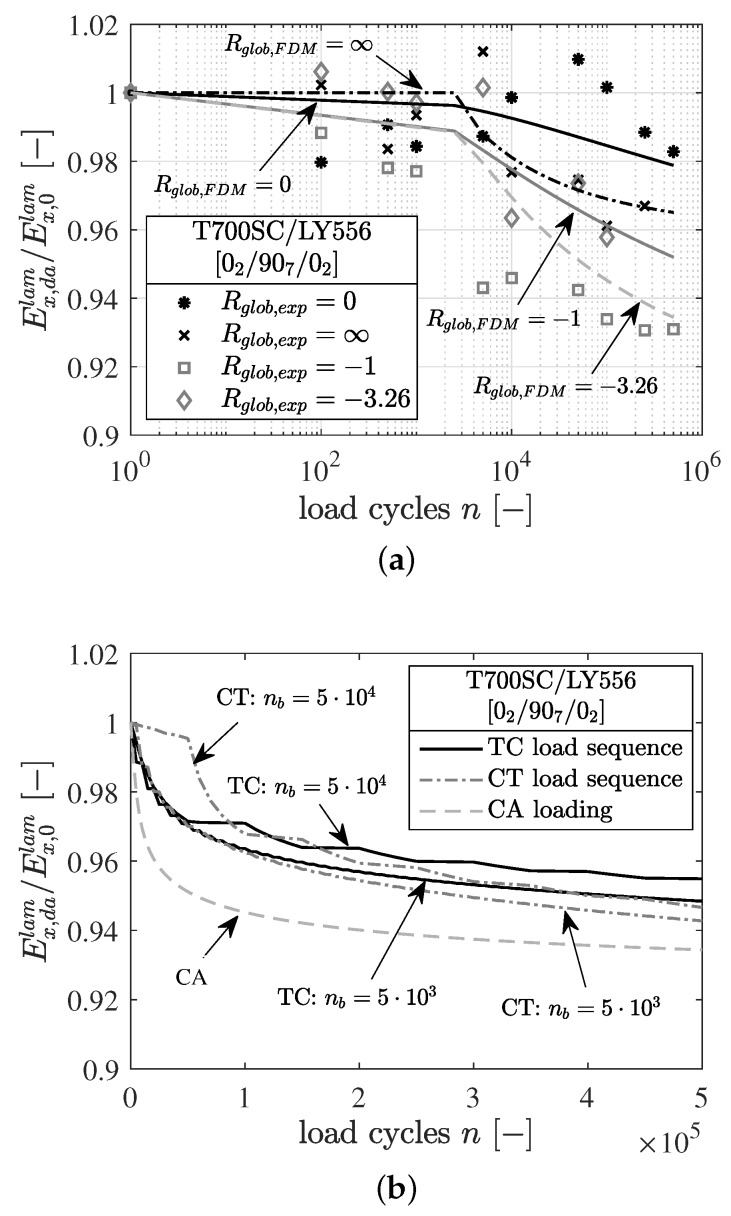
(**a**) Numerical prediction (subscript FDM) and comparison with experiments (subscript exp) of constant amplitude loading (CA) at different global stress ratios: $R_{glob}^{lam}=\left\{0,-1,-3.26\right\}$ with $\sigma_{max}^{lam}=105$ MPa and $R_{glob}^{lam}=\infty$ with $\sigma_{min}^{lam}=-380$ MPa; (**b**) Numerical prediction of block-loading with different load block length and load sequences: tension-compression sequence (TC) with $n_{b}=\left\{5\times 10^{3},\;5\times 10^{4}\right\}$ and compression-tension sequence (CT) with $n_{b}=\left\{5\times 10^{3},\;5\times 10^{4}\right\}$, CA with $R_{glob}^{lam}=-3.26$ and $\sigma_{max}^{lam}=105$ MPa.

The calculation results for the various stress ratios under CA loading are depicted and compared in Figure 15a. The degradation of the normalized laminate stiffness $E_{x,da}^{lam}/E_{x,0}^{lam}$ is represented depending on the number of load cycles *n*. Therefore, the degraded laminate module $E_{x,da}^{lam}$ is calculated according to Equation (9).

(9) $$E_{x,da}^{lam}=\frac{1}{t^{lam}}\left[t^{90}E_{da}^{90}+t^{0}E_{da}^{0}\right].$$

The simulations are carried out with a block length of $n_{b}=2500$ cycles and a maximum number of cycles of $n=5\times 10^{5}$. It can be clearly seen that the numerically predicted fatigue damage evolution is more pronounced for global alternating loading ($R_{glob}^{lam}=\left\{-1,-3.26\right\}$) than for global pulsating loading ($R_{glob}^{lam}=\left\{0,\infty \right\}$). Depending on the amount of the stress amplitude, as well as the change of the local stress ratios $R_{loc}$ caused by the residual stresses, the damage state at the end of the calculation is more pronounced for local alternating stresses when comparing positive ($E_{x,da}^{lam}\left(R_{glob}^{lam}=\infty /R_{loc}^{90}=-1.5\right)<E_{x,da}^{lam}\left(R_{glob}^{lam}=0/R_{loc}^{90}=0.6\right)$) and negative global stress ratios ($E_{x,da}^{lam}\left(R_{glob}^{lam}=-3.26/R_{loc}^{90}=-0.8\right)<E_{x,da}^{lam}\left(R_{glob}^{lam}=-1/R_{loc}^{90}=0.2\right)$). When comparing the numerical results to the experimental observations conducted in Just [7], a good agreement is achieved and the prediction quality of the FDM under CA loading is shown.

In the second part of this study, the influence of load reversals at block-loading is investigated. Figure 15b shows the simulation results of the damage evolution under block-loading. Based on the experimental investigations in Section 2, the block lengths $n_{b}$, global stress ratios $R_{glob}$, as well as the maximum and minimum stress levels, are selected as input variables for the numerical calculations. The results include comparisons of load sequence investigations of short ($n_{b}=5\times 10^{3}$) with long ($n_{b}=5\times 10^{4}$) load blocks, of TC with CT sequences and of block-loading with CA loading (cf. Figure 2).

When comparing short with long load blocks, the FDM calculates a marginally higher damage for short load blocks. This effect is visible in both TC and CT sequences. The deviation of the damage state in both cases (TC, CT) is less than one percent after $5\times 10^{5}$ load cycles. Comparing the damage evolution of TC with CT sequences, the FDM predicts a higher amount of damage with CT sequences. However, the deviation here is similarly small as in the case of comparing short and long load blocks. If, on the other hand, the block loads described are compared with the CA loading, a clear difference can be seen in the respective damage evolution. In the case of CA loading, the damage evolution is significantly higher in consequence of the higher load amplitude and the alternating load occurring over the entire loading.

A similar trend of damage evolution can be observed by comparing the obtained simulation results with the crack density evolution observations from Section 2, see Figure 3d. The damage states of the different predicted load sequences (TC, CT as well as with long and short load blocks) show comparable results after $5\times 10^{5}$ load cycles. Similar to the crack counting analysis in Section 2.2, it can be seen that the influence of pure compression damage components on the damage development is marginal. The predicted damage induced under compression is attributable to the tension-induced damage caused by the local alternating load in the embedded 90${}^{\circ }$-layer due to residual stresses after curing.

## 5. Conclusions

The present collaborative study provides experimental results on the damaging process of CFRP cross-ply laminates under constant amplitude and block-loading conditions, mathematical methods for describing the mesoscopic degradation as well as the mesoscopic damage behavior and micromechanical explanations for the observed behavior based on neat resin experiments and RVE simulations for constant and fatigue loading.

The results for block-loading experiments for TC and CT sequences, respectively, did not show significant differences in terms of crack formation and delamination growth. However, compared to fully reversed constant amplitude tests with the same stress levels, the block-loading experiments showed lower crack densities and less delamination growth. Accompanied resin creep and fatigue experiments in tension revealed a tendency for increasing stiffness and strength of the matrix over time with the potential of influencing the damage behavior at different stress ratios.

Simulation with micromechanical RVE-model showed similar strain distribution in matrix when the FRP is loaded in different sequences with the same stress levels and therefore support the experimental findings. It was consistently found that only by considering residual stresses the mesoscopic damage behavior at different stress ratios can be interpreted. It has been shown additionally that residual stresses change significantly over time. Furthermore, the differences in the cracking behavior are also attributed to the range of the strain energy release rate for different load ratios considering residual stresses. By calculating the damage-driving range of ERR with respect to the changing residual stresses, the authors were able to pool the different sets of experiments into one Paris-like crack growth relation.

The experimental findings are further on incorporated to the energy-based FDM. The user-defined material subroutine, proposed for fatigue damage analysis on meso- and macro-scale levels, was extended for the analysis of variable amplitude loading and the consideration of manufacturing-induced residual stresses. It was successfully shown that load sequence effects in the context of load reversals can be reproduced with the flexible modeling approach. As seen from the experiments, the various TC load sequences examined showed a very similar damage evolution. In comparison to a constant TC alternating load, the prediction with load sequences results in a significantly accelerated increase in damage.

## Figures and Tables

**Table 1:** Elastic, strength and thermal properties of T700SC/LY556 composite material [7].

$E_{\|}^{t}$	$E_{\|}^{c}$	$E_{⊥}^{t}$	$E_{⊥}^{c}$	$G_{\|⊥}$	$\nu_{\|⊥}$	$T_{g}$
(GPa)	(GPa)	(GPa)	(GPa)	(GPa)	(-)	(${}^{\circ }$C)
129.4	110.7	8.05	8.87	3.91	0.317	146.4
±5.4	±21.7	±0.44	±0.20	±0.19	±0.0076	±2.4
$R_{\|}^{t}$	$R_{\|}^{c}$	$R_{⊥}^{t}$	$R_{⊥}^{c}$	$R_{\|⊥}$	$\alpha_{\|}^{*}$	$\alpha_{⊥}^{*}$
(MPa)	(MPa)	(MPa)	(MPa)	(MPa)	($10^{-6}/$K)	($10^{-6}/$K)
2089	1032	36.2	164.4	52.2	0.5	38
±53	±221	±5.3	±7.9	±5.4	-	-

${}^{t}$—tension, ${}^{c}$—compression, ${}_{\|}$—parallel to fiber direction, ${}_{⊥}$—transverse to fiber direction, ${}^{*}$—mean value (RT—140 ${}^{\circ }$C).

**Table 2:** Parameters of the modified van’t Hoff equation and Prony series for LY556 resin.

*B*	*C*	$T_{m}$	$T_{0}$	*K*	$G_{\infty }$	$G_{i}$	$\tau_{i}$
(-)	(-)	(K)	(K)	(GPa)	(GPa)	(GPa)	(s)
0.16667	0.0001188	296.15	296.15	3888.9	1295.35	0.0475	cf. Figure 7

**Table 3:** Overview of the local maximum and minimum stress components ($\sigma_{max}^{90}$ and $\sigma_{min}^{90}$) and their associated local stress ratios $R_{loc}^{90}$ as a result of the manufacturing-induced residual stress field in the $90^{\circ }$-layer at different global applied stress ratios $R_{glob}^{lam}$ on the laminate.

$R_{\mathrm{glob}}^{\mathrm{lam}}$	$\sigma_{\max}^{90}$	$\sigma_{\min}^{90}$	$R_{\mathrm{loc}}^{90}$
(-)	(MPa)	(MPa)	(-)
0	41.3	22.8	0.6
∞	22.5	−34.9	−1.5
−1	41.3	7.0	0.2
−3.26	41.3	−34.8	−0.8
